# The Discrepancy Between External and Internal Load/Intensity during Blood Flow Restriction Exercise: Understanding Blood Flow Restriction Pressure as Modulating Factor

**DOI:** 10.1186/s40798-024-00759-9

**Published:** 2024-09-04

**Authors:** Robert Bielitzki, Martin Behrens, Tom Behrendt, Alexander Franz, Christoph Centner, Luke Hughes, Stephen D. Patterson, Johnny Owens, Michael Behringer, Lutz Schega

**Affiliations:** 1 Department of Sport Science, Institute III, Otto-von-Guericke University Magdeburg, Magdeburg, Germany; 2 University of Applied Sciences for Sport and Management Potsdam, Potsdam, Germany; 3 Department of Orthopedics and Trauma Surgery, University Hospital Bonn, Bonn, Germany; 4 Department of Sport and Science, University of Freiburg, Freiburg, Germany; 5 Department of Sport, Exercise and Rehabilitation, Northumbria University, Newcastle, UK; 6 Faculty of Sport, Technology and Health Science, St Mary’s University, Twickenham, London, UK; 7 Clinical Education Owens Recovery Science, San Antonio, TX USA; 8 Department of Sports Sciences, Goethe University Frankfurt, Frankfurt a. M., Germany

**Keywords:** Vascular occlusion, Metabolic stress, Muscle pain, Effort perception, Stimulus, Terminology

## Abstract

Physical exercise induces acute psychophysiological responses leading to chronic adaptations when the exercise stimulus is applied repeatedly, at sufficient time periods, and with appropriate magnitude. To maximize long-term training adaptations, it is crucial to control and manipulate the external load and the resulting psychophysiological strain. Therefore, scientists have developed a theoretical framework that distinguishes between the physical work performed during exercise (i.e., external load/intensity) and indicators of the body's psychophysiological response (i.e., internal load/intensity). However, the application of blood flow restriction (BFR) during exercise with low external loads/intensities (e.g., ≤ 30% of the one-repetition-maximum, ≤ 50% of maximum oxygen uptake) can induce physiological and perceptual responses, which are commonly associated with high external loads/intensities. This current opinion aimed to emphasize the mismatch between external and internal load/intensity when BFR is applied during exercise. In this regard, there is evidence that BFR can be used to manipulate both external load/intensity (by reducing total work when exercise is performed to exhaustion) and internal load/intensity (by leading to higher physiological and perceptual responses compared to exercise performed with the same external load/intensity without BFR). Furthermore, it is proposed to consider BFR as an additional exercise determinant, given that the amount of BFR pressure can determine not only the internal but also external load/intensity. Finally, terminological recommendations for the use of the proposed terms in the scientific context and for practitioners are given, which should be considered when designing, reporting, discussing, and presenting BFR studies, exercise, and/or training programs.

## Key Points

- The application of BFR during exercise with low external load/intensity can lead to internal responses that are commonly associated with high external load/intensity resulting in a discrepancy between the characteristics of exercise and the acute psychophysiological responses.
- The BFR pressure can be adjusted to increase the internal load/intensity (i.e., elevating physiological and perceptual responses) to intensify the exercise stimulus or to decrease the external load/intensity (e.g., reducing the number of repetitions when exercise is performed to exhaustion), which is of particular importance during musculoskeletal rehabilitation when high or cumulative low mechanical stress might be contraindicated.
- We encourage researchers to adapt their wording in the BFR literature accordingly, given that the extent of internal load/intensity during BFR exercise is determined by the interaction of several external exercise variables (e.g., external resistance, number of repetitions/cycles, cuff pressure) to specify the generated exercise stimulus (e.g., “low external load BFR walking”, “low external load BFR resistance exercise”).

## Introduction

Over the last two decades, blood flow restriction (BFR) training has increasingly been used to improve performance across different populations (e.g., elite athletes [1], healthy active elderly [2], and patients during musculoskeletal rehabilitation [3]). In this regard, BFR has been applied during several exercise modalities such as resistance [4], endurance [5], balance [6], or whole-body vibration exercise [7]. To achieve a target restriction pressure, usually a pneumatic tourniquet cuff is applied to the proximal part of the limb to partially restrict and completely occlude arterial inflow and venous return of the blood, respectively [8]. It is known that the degree of restriction of arterial and venous blood flow induced by the applied cuff depends on various moderator variables including individual characteristics (e.g., blood pressure, arm circumference [9]), and cuff properties (e.g., width [10], stiffness [11]). To account for these moderator variables, the target pressure is commonly determined as a percentage of the arterial occlusion pressure (AOP; also referred to as limb occlusion pressure), which is defined as the lowest pressure that is required to occlude arterial inflow to the limb [12]. The external pressure generated by the BFR cuff promotes blood pooling, which induces a local hypoxic environment distal to the restriction [13]. It is assumed that the hypoxia-induced shift towards a greater proportion of anaerobic metabolism and the reduced removal of metabolites from the muscle produced during exercise leads to an increased metabolic stress and performance decline (i.e., motor performance fatigue [14]) compared to the same exercise without BFR [15]. When applying BFR, it is recommended to use 20–40% of the one-repetition-maximum (1RM) and < 50% of peak oxygen uptake (${\dot{{\text{V}}}}{{\text{O}}}_{2}$ peak) or heart rate reserve for resistance and aerobic exercise, respectively [16]. In this regard, the terms low-load BFR exercise [17–19] or low-intensity BFR exercise [20, 21] are frequently used in the literature. However, the external load/intensity is characterized by the exercise characteristics (e.g., external resistance, repetition scheme, volitional muscle failure), while the internal load/intensity (i.e., psychophysiological responses) is mirrored by multiple variables including heart rate or rate of perceived exertion (RPE) [22–24]. In this context, the application of BFR during low load/intensity exercise and its mode of action can lead to psychophysiological responses that are typically not associated with low but moderate or even high external load/intensity exercise. Therefore, the aim of this opinion article is (i) to elaborate on the interaction between external and internal load/intensity moderated by the level of BFR and its relevance for researchers and practitioners as well as (ii) to discuss the potential of BFR as an additional variable to manipulate external and internal load/intensity when designing exercise and training programs [25].

## Defining External and Internal Load/Intensity

Physical activity and/or exercise (e.g., running, cycling, swimming) triggers acute psychophysiological responses and can lead to chronic adaptations when the exercise stimulus is applied repetitively, at sufficient time periods, and with appropriate magnitude [23, 26]. To maximize long-term training adaptations, it is crucial to control and manipulate the stress applied to the exercising individual and the resulting psychophysiological strain. Therefore, scientists have developed theoretical frameworks that distinguish between the physical work performed during exercise (i.e., external load/intensity) as well as indicators of the body’s psychophysiological reactions and the strain experienced by specific tissues (i.e., internal load/intensity) in response to the applied external training load/intensity [22–24, 27]. The terminology ‘external and internal training load’ was recently criticized from a biomechanical perspective pointing out that load is a mechanical variable, which describes forces [28–30]. However, it was subsequently argued that mechanics do not have the monopoly on the term ‘load’ or other common terms like ‘stress’ and ‘fatigue’. Consequently, ‘training load’ must be considered a label representing a higher order construct with subdimensions (i.e., referred to as external and internal load from this point) that provides a framework to support the research and practical field [31]. The distinction between external and internal load allows for a better understanding of the training process and the associated exercise-related adaptations (Table 1). The external load is determined by the characteristics of exercise (i.e., physical work performed during exercise) and the measures to quantify the external load depend on the exercise modality (e.g., endurance and resistance exercise) and/or sport (e.g., team sports). For instance, during endurance exercise, the external load is determined, e.g., by the velocity, power output or total work, exercise duration, distance covered, and rest intervals. Similar metrics are used to quantify the external load in team sports (e.g., velocity, distance covered, accelerations), while the external resistance, time under tension, number of repetitions per set, number of sets, and rest intervals are often used to describe the external load during resistance exercise [23, 29, 32, 33]. To cope with the external load, acute and individual psychophysiological responses are initiated that depend on the exercise modality (e.g., endurance or resistance exercise) and specific contextual factors (e.g. personal and environmental factors) [23, 34]. Therefore, exercise-specific variables are used to describe the internal load. For instance, heart rate (HR) is often used to characterize the internal load during endurance exercise, although this marker is often not a suitable internal load measure for resistance exercise [23]. Besides physiological markers, perceptual responses during exercise (e.g., RPE or effort perception, exercise-induced muscle pain perception) can be used to characterize the internal load during several exercise modalities [33–36]. Given that the psychophysiological responses are strongly determined by the characteristics of the performed exercise [14], the respective internal load markers scale with the applied external load measures. Nevertheless, due to modifiable and non-modifiable personal factors (e.g., nutrition, training status, health, psychological status, genetics) that affect the extent of the individual psychophysiological response to exercise, the same external load (e.g., same running velocity) generates interindividual differences in internal load markers (e.g., HR, RPE). Moreover, modifiable personal factors (e.g., nutrition, training status) and environmental conditions (e.g., heat [37], local and systemic hypoxia [38]) can change, leading to different psychophysiological responses to the identical exercise stimulus in the same individual. Given that the interplay of the characteristics of the exercise, the contextual factors, and the resulting acute response to the exercise determine chronic adaptations and, thus, the training outcome, it is recommended to use internal load markers in conjunction with external load measures to monitor and control the training process [23, 39–42]. In addition, specific contextual factors should also be considered, especially if the training method used implies a deliberate change in one or more of these factors (e.g., hypoxia [43] or heat conditioning [44]).

In contrast to the general observation that performing exercise with higher external loads (e.g. 70% 1RM) [45] or until exhaustion [46] results in an increased internal load, similar high psychophysiological responses are also present when exercising with low external loads (e.g., 30% 1RM) combined with BFR. For instance, studies have found a similar RPE [47, 48] and discomfort [49, 50] for resistance exercise at ≤ 30% 1RM with BFR (65–75 repetitions) and resistance exercise at ≥ 70% 1RM without BFR (30–40 repetitions). Moreover, when BFR is applied during specific exercise modalities (e.g., repeated cycling sprints or resistance exercise performed to exhaustion) not only the internal but also the external load can be manipulated due to an accelerated motor performance fatigue development and the subsequent reduction in external load measures (i.e., power output [25], number of repetitions [51]). Therefore, an adequate exercise and training prescription should primarily focus on the internal load, while also considering external load and relevant contextual factors (i.e., modifiable and non-modifiable personal factors and environmental conditions) [27, 34].

**Table 1:** Definition and exercise-dependent measures of external and internal load as well as contextual factors during blood flow restriction (BFR) exercise

	**External load**	**Internal load**	**Contextual factors**
Definition	Physical work performed during exercise	Acute psychophysiological responses	Modifiable and non-modifiable determinants
Characteristics and measures	Resistance e.g., external resistance, time under tension, number of repetitions per set, number of sets, rest intervals	Resistance e.g., effort perception, exercise-induced muscle pain perception	Personal factors e.g., training status, age, sex Environmental factors e.g., climatic and geographic conditions BFR-related factors e.g., cuff pressure^1^, type of application^2^
	Endurance e.g., velocity, power output or total work, exercise duration, distance covered, rest intervals	Endurance e.g., heart rate, blood lactate concentration, effort perception, exercise-induced muscle pain perception	
	Team sports e.g., velocity, distance covered, accelerations	Team sports e.g., heart rate, blood lactate concentration, effort perception, exercise-induced muscle pain perception	

^1^percentage arterial occlusion pressure

^2^continuous or intermittent (i.e., no BFR during rest or exercise)

## The Discrepancy Between Internal and External Load during Blood Flow Restriction Exercise

### Internal load Measures in Response to Exercise with Low External Load Combined with and without Blood Flow Restriction

**Fig. 1:**
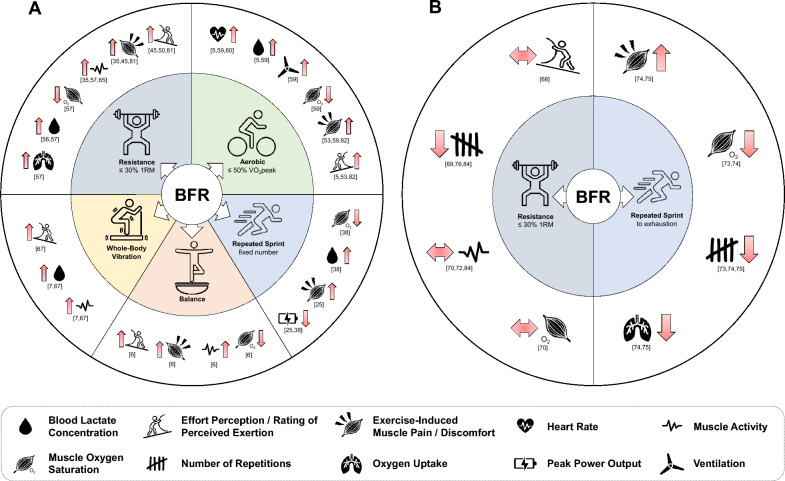
Schematic illustration of the influence of blood flow restriction (BFR) on example parameters of internal and external load during several exercise modalities with (**A**) matched external load and (**B**) to exhaustion. The numbers on the individual parameters refer to the articles’ reference list

When combining BFR with exercise using low external loads, the psychophysiological responses representing the internal load can increase compared to exercise without BFR (Fig. 1A). Therefore, the relationship between exercise (e.g., slow to fast running or cycling) and the intensity categories (i.e., light, moderate, vigorous, high [52]) does not apply for BFR exercise resulting in a potential discrepancy between external and internal load measures. A recent meta-analysis [46] revealed that when performing resistance exercise with identical external load at ≤ 30% 1RM, participants’ perceptual responses (e.g., effort and exercise-induced muscle pain/discomfort perception) were higher with BFR than without BFR. For instance, Miller et al. [50] reported higher RPE and muscle discomfort during leg press and knee extension exercise at 30% 1RM with BFR (50% AOP) compared to the same exercise (i.e., identical external load) without BFR. Similar results were found by Mok et al. [53] revealing higher leg discomfort during 5 walking intervals of 2 min at 5 km·h^−1^ with BFR (200 mmHg) compared to walking alone.

The different perceptual responses can be explained by accompanying physiological changes associated with BFR exercise. For example, given that exercise-induced muscle pain or discomfort perception is triggered by the stimulation of nociceptive group III and IV muscle afferents, venous blood pooling (induced by the external cuff pressure during BFR) might lead to venous expansion, which has been shown to stimulate group IV afferents in an animal study [54]. Moreover, nociceptive muscle afferents seem to be sensitive to high amounts of metabolites [55], which are associated with BFR exercise due to an increased anaerobic metabolism and impaired metabolite removal. In this regard, invasive catheter examinations by Franz et al. [56] have revealed that BFR (50% AOP) induced elevated venous blood lactate concentration (BLC) and increased metabolites (i.e., K^+^, Ca^2+^, Na^+^) leading to metabolic acidosis (i.e., lower arterial and venous pH) during 4 sets (75 repetitions) of unilateral biceps curls at 30% 1RM compared to the same exercising without BFR. Moreover, studies have shown, for instance, higher deoxyhemoglobin concentration as a proxy of metabolic stress during 4 sets (75 repetitions) of isometric knee extensions at 20% MVC [57], as well as increased muscle thickness as a marker for hydration-mediated cell swelling during 4 sets (75 repetitions) of unilateral leg press at 30% 1RM [58] performed with BFR compared to the same exercise without BFR. In this context, Kilgas et al. [59] found higher changes in BLC and quadriceps muscle deoxyhemoglobin concentration accompanied by higher exercise-induced muscle pain ratings during 6 cycling intervals of 2 min at 40% ${\dot{{\text{V}}}}{{\text{O}}}_{2}$ peak when using BFR (60% and 80% AOP) compared to exercising at the same external load without BFR.

The higher RPE or effort perception during BFR resistance [50] and endurance exercise [60] might be related to the loss of contractile function induced by the greater metabolic disturbance [61, 62] due to impaired removal of accumulated metabolites leading to decrements in Ca^2+^-sensitivity and/or release from the sarcoplasmic reticulum [63]. Based on the corollary discharge model discussed by Pageaux [64], the central motor command increases as a compensatory mechanism to counteract the loss of contractile function due to greater metabolic stress. The higher descending neural drive to the muscle (i.e., increase in muscle activity) is required to maintain the muscle forces needed for exercise continuation [35, 65, 66] resulting in a higher effort perception. In this context, Husmann et al. [35] showed that the application of BFR (60% AOP) induced higher ratings of effort perception accompanied by greater muscle activity in the vastus medialis and lateralis muscles during knee extension exercise at 30% 1RM compared to without BFR. These results are supported by Cai et al. [67] who found higher rectus femoris and vastus lateralis muscle activity as well as higher RPE averaged over 10 sets of whole-body vibration exercise in a squat position with BFR (140 mmHg) compared to identical exercise alone.

### External Load Measures in Response to Exercise with and without Blood Flow Restriction Performed to Exhaustion

Furthermore, studies have shown a lower external load (i.e., lower number of repetitions) and similar perceptual responses during BFR compared to without BFR when the exercise was performed to exhaustion [68, 69] (Fig. 1B). In this regard, Kolind et al. [70] found that participants who performed one set of unilateral knee extensions at 20% 1RM with BFR (100 mmHg) achieved 43% fewer repetitions with higher exercise-induced muscle pain perception. However, although the number of repetitions was lower in the BFR condition, similar changes in muscle oxygenation and muscle activity of vastus medialis and lateralis were found at the respective percentage of time to exhaustion [70]. Comparable to Kolind et al. [70], Behringer et al. [71] found that BFR (AO−20 mmHg) reduced the number of repetitions during four sets of unilateral eccentric knee extensions at 75% 1RM, while inducing similar internal responses (e.g., peak BLC, insulin-like growth factor 1, creatine-kinase, muscle pain). Furthermore, Buckner et al. [72] also observed a reduced number of repetitions to exhaustion during 4 sets of unilateral elbow flexions at 15% 1RM with BFR (80% AOP) compared to without BFR. Therefore, the additional application of BFR during exercise performed to exhaustion can reduce repetitions and time to exhaustion and thus the external load, while eliciting similar levels of internal responses (e.g., blood pooling, muscle oxygenation, and muscle activity at the same percentage of exercise time) [70]. Furthermore, BFR was also shown to reduce the external load during maximal motor tasks meaning that multiple studies have revealed a reduced number of total sprints during repeated-sprint exercise to exhaustion [73–75]. This phenomenon might be explained by the accelerated motor performance fatigue development [14] induced by BFR, which was shown by Husmann et al. [35] who found a larger decline in maximal voluntary torque in the BFR (60% AOP) compared to non-BFR condition after each set during 4 sets (75 repetitions) of knee extensions at 30% 1RM. In addition, Behrendt et al. [38] found that BFR (40% AOP) led to a greater decline in mean and peak power output during 6 × 10 s repeated cycling sprints compared to the same exercise without BFR. The lower external load during each training session consequently results in a reduced external training load during an intervention period, as shown by Pignanelli et al. [76]. The authors found a reduction in external load (i.e., training volume) of ~ 33% during a 6-week BFR training period (60–70% AOP) using single-leg squats at 30% 1RM to volitional failure. Interestingly, despite a lower training volume, similar increases in muscle strength and size were found. This is of particular importance for musculoskeletal rehabilitation during which gains in muscle strength and size are desired but a high or cumulative low mechanical stress might be contraindicated [77].

## Understanding Blood Flow Restriction Pressure as a Determinant to Modulate Internal and External Load

The current BFR literature describes the cuff pressure for resistance and endurance exercise to be sufficient and beneficial between 40 and 80% AOP [16]. Accordingly, the level of cuff pressure is a critical variable to manipulate the psychophysiological responses to BFR exercise [78–80]. For instance, Ilett et al. [57] investigated the physiological responses during 4 sets of isometric knee extensions (32 repetitions) at 80% MVC (i.e., high external load) and 4 sets (75 repetitions) at 20% MVC (i.e., low external load) combined with BFR at 80%, 60%, and 40% AOP as well as without BFR. The authors found similar BLC levels for high external load exercise without BFR and low external load exercise combined with BFR at 80% AOP as well as for low external load exercise combined with BFR at 40% AOP and without BFR. In addition, Hughes et al. [81] found a similar RPE during 4 sets of unilateral leg press exercise (75 repetitions) at 30% 1RM combined with 40% AOP, but a higher RPE when combined with 80% AOP, compared with 3 sets (30 repetitions) at 70% 1RM. In another study by Hughes et al. [82], muscle discomfort was found to be higher during 20 min of aerobic cycling at 40% ${\dot{{\text{V}}}}{{\text{O}}}_{2}$ peak with BFR at 80% AOP compared to 40% AOP. Furthermore, Loenneke et al. [48] found higher ratings of discomfort at 60% AOP compared with 40% AOP during 4 sets (75 repetitions) of bilateral knee extensions at 20% 1RM combined with BFR. The perceptual differences might be related to the higher mechanical pressure on the blood vessels leading to a greater extent of metabolic disturbances (e.g., higher deoxyhemoglobin concentration, increased venous blood pooling and expansion, greater metabolite accumulation) finally causing, for instance, higher muscle pain ratings due to greater activation of group III and IV afferent fibers [55]. This assumption is supported by the results of Bielitzki et al. [6], who revealed that effort perception, exercise-induced leg muscle pain perception, and myoelectrical activity of the quadriceps muscle (recorded via surface electromyography) were higher, while muscle oxygen saturation in the vastus lateralis muscle was lower during the last set of a static BFR balance exercise with 80% AOP compared to 40% AOP. These findings are similar to those by Ilett et al. [57], who revealed that BLC, HR, and muscle activity were higher, while muscle oxygen saturation was lower, when applying BFR at 80% AOP compared to 40% AOP during 4 sets (75 repetitions) of rhythmic isometric knee extensions at 20% MVC. Therefore, it can be assumed that physiological and perceptual responses during exercise with low external load combined with BFR depend, among others, on the applied cuff pressure [6, 57, 59, 81, 82].

Regarding the manipulation of external load, a recent meta-analysis by Cerqueira et al. [83] revealed that high cuff pressures are required to reduce the time to exhaustion and, thus, the external load. Therefore, the cuff pressure seems not only to modulate the internal load during volume-matched exercise but also the external load, when exercises are performed to exhaustion. For example, Jessee et al. [84] revealed lower repetitions to volitional failure during 4 sets of unilateral knee extensions at 15% 1RM with BFR at 80% AOP (mean: 73 repetitions) compared to 40% AOP (mean: 114 repetitions), while rectus femoris and vastus lateralis muscle activity in each set were similar between pressures. Comparable findings were revealed by Buckner et al. [72] during 4 sets (performed to exhaustion) of elbow flexion at 15% 1RM with BFR at 80% AOP and 40% AOP.

Of note, a recent study by Jacobs et al. [85] found that pneumatic tourniquet systems with autoregulation (i.e., cuff pressure adapts automatically to changes in limb circumference during exercise) have led to lower RPE and discomfort during 4 sets (75 repetitions) as well as a lower number of repetitions per set during 4 sets (to exhaustion) of unilateral knee extension at 20% 1RM with 60% AOP compared a non-autoregulated system. Therefore, cuff type (e.g., single- vs. multi-chambered bladder) and type of tourniquet system (e.g., non-auto- vs. autoregulated) might additionally influence the internal and external load [86].

**Fig. 2:**
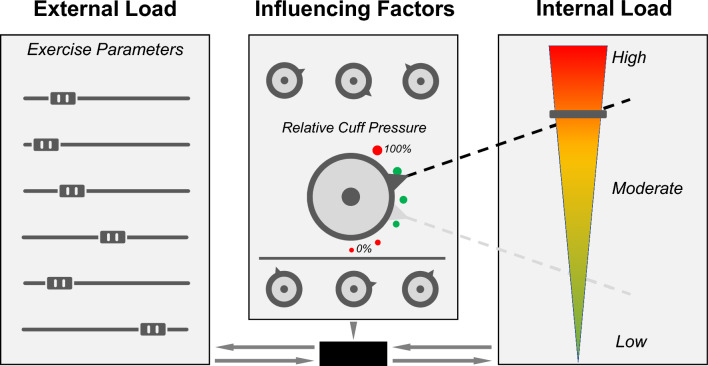
Illustration of a conceptual framework of exercise with blood flow restriction. The external load is determined by a variety of exercise parameters, which also dictate the psychophysiological responses, and therefore, the internal load. The internal load in response to a specific external load depends on a multitude of influencing factors (i.e., environmental and personal factors). The level of relative cuff pressure, along with other variables, represents a modifiable environmental factor to manipulate the internal load. Relative cuff pressures between 40 and 80% of the individuals’ arterial occlusion pressure are assumed to induce favorable long-term adaptations

In summary, current evidence indicates that the amount of relative cuff pressure (i.e., %AOP) can be used to manipulate physiological and perceptual responses during resistance [48, 79, 81], endurance [82], and balance exercise [6] as well as neuromuscular electrical stimulation [87] when the external load is kept constant (Fig. 2). The amount of BFR pressure is of particular importance especially when only very low external loads with a fixed number of repetitions are applied (e.g., ≤ 20% 1RM [78, 80], static balance exercise [6]). This might be relevant for individuals that are only able to tolerate very low external loads (e.g., during musculoskeletal rehabilitation). Therefore, practitioners (e.g., physicians and therapists) should be aware of the BFR pressure as an additional variable to manipulate psychophysiological responses during exercise. On the one hand, by elevating the cuff pressure, the stimulus during exercise can potentially be intensified by increasing physiological responses (e.g., BLC, deoxygenation, muscle activity) as well as reducing the cumulative external load (e.g., number of repetitions) when exercising to exhaustion. On the other hand, if participants or patients are less tolerant to pain (i.e., exercise-induced muscle pain, cuff pressure-induced discomfort), the BFR pressure can be decreased to lower the local hypoxic stimulus and support metabolite removal in order to lower perception of effort and pain. Since it has been found that perceptual responses to exercise are significant predictors of future physical activity behavior [88], reducing the applied BFR pressure might ensure exercise adherence. However, as with other forms of exercise, chronic exposure to BFR may attenuate perceptions of effort and pain thus reducing internal load and creating a window to increase external load [89].

## Conclusion

The additional application of BFR during exercise can, on the one hand, increase the internal load (i.e., physiological and perceptual responses to exercise) when the external load (i.e., physical work performed during exercise) is similar. On the other hand, applying BFR during specific exercise modalities (e.g., resistance exercise to exhaustion or repeated sprint exercise) can reduce the external load (i.e., number of repetitions, power output) by accelerating motor performance fatigue development without substantially different psychophysiological responses (i.e., internal load). Of note, there are several other aspects that influence internal and external loads during BFR exercise (e.g., continuous vs. intermittent BFR [90, 91]). Furthermore, the amount of cuff pressure applied during exercise can be used to manipulate internal and external loads to maximize long-term training adaptations and adherence.

### Recommendations for Scientists

This opinion aimed to encourage scientists in the field of BFR research to use the established terminology external and internal load [23, 25] to describe the characteristics of the applied exercise protocol and the associated psychophysiological changes. Adopting this terminology may allow a more precise classification of the measured outcomes and a better understanding of the interactions between external and internal loads during BFR exercise mediated by the level of cuff pressure. Given that the interaction of several external exercise parameters (e.g., external resistance, number of repetitions/cycles, cuff pressure) determine the extent of internal load during BFR exercise, researchers are encouraged to specify the wording in their articles accordingly to clarify whether the external or internal load was low or high in their BFR studies. For instance, resistance exercise at ≤ 30% 1RM combined with BFR can lead to high perceptual responses (i.e., high internal load) and might benefit from a specification in wording by using the description “low external load BFR resistance exercise” instead of “low load BFR resistance exercise”. The integration of the terms external and/or internal load in the wording can be a helpful addition for the readers to instantly get a clearer view on the exercise characteristics and/or the psychophysiological response.

### Recommendations for Practitioners

Practitioners should be aware of the differentiation between external as well as internal load and should consider the BFR pressure as an additional exercise variable when designing exercise and training programs (also with regard to the used cuff type and tourniquet system). The exercise/training stimulus can potentially be increased by elevating the cuff pressure. Furthermore, reducing the cuff pressure lowers effort and exercise-induced muscle pain perception, which is of particular importance for participants or patients with pain intolerance to increase exercise adherence. In addition, the external load (e.g., number of repetitions during exercise) can be reduced by adding high BFR pressures [83], while inducing similar internal responses. This could be of particular importance during musculoskeletal rehabilitation, when high as well as cumulative low mechanical loads may be contraindicated.

## Data Availability

Not applicable.

## Abbreviations

1RM: One-repetition-maximum
AOP: Arterial occlusion pressure
BLC: Blood lactate concentration
BFR: Blood flow restriction
HR: Heart rate
MVC: Maximal voluntary contraction
RPE: Rate of perceived exertion
$${\dot{\text{V}}}{{\text{O}}}_{2}$$: Oxygen uptake
